# Stereotactic ablative radiotherapy (SABR) for recurrent and previously irradiated head and neck cancers

**DOI:** 10.1259/bjro.20190051

**Published:** 2020-03-03

**Authors:** Anil Kumar Anand, Bharat Dua, Anil Kumar Bansal, Heigrujam Malhotra Singh, Amit Verma, Amit Kumar

**Affiliations:** 1 Department of Radiation Oncology, Max Institute of Cancer Care, Max Super Speciality Hospital, Saket, New Delhi, India; 2 Division of Medical Physics, Department of Radiation Oncology, Max Institute of Cancer Care, Max Super Speciality Hospital, Saket, New Delhi, India; 3 Division of Molecular Oncology, Max Institute of Cancer Care, Max Super Speciality Hospital, Saket, New Delhi, India; 4 Department of Imaging, Max Super Speciality Hospital, Saket, New Delhi, India

## Abstract

**Objective:**

To assess the response and toxicity of stereotactic ablative radiotherapy (SABR) in patients with recurrent head and neck cancer (HNC), who had previously received radiation for their primary tumor.

**Methods:**

Between 2014 and 2018, patients who received SABR to recurrent HNC within the previously irradiated region were retrospectively reviewed. Mean age was 60 years (range 30–78 Years). Histology was confirmed in all patients. MRI and /or CT-positron emission tomography were done to evaluate local extent and to rule out metastasis. Response was assessed as per RECIST/PERCIST Criteria. Cox proportional hazards regression and the Kaplan–Meier methods were used for statistical analysis.

**Results:**

32 patients received SABR. RPA Class II, III patients were 20 and 12 respectively. 87% patients received a dose of ≥30 Gy/5 fractions. Median follow-up was 12 months. Estimated 1 year and 2 years local control was 64.2 and 32% and 1 year and 2 years overall survival was 67.5 and 39.5% respectively. Acute Grade 2 skin and Grade 3 mucosal toxicity was seen in 31.3 and 28% patients respectively. Late Grade 3 toxicity was seen in 9.3% patients.

**Conclusion:**

Re-irradiation with SABR yields high local control rates and is well tolerated. It compares favorably with other treatment modalities offered to patients with recurrent HNC. It is also suitable for patients of RPA Class II and III. There is need for novel systemic agents to further improve the survival.

**Advances in knowledge:**

Treatment of patients with recurrent HNC is challenging and is more difficult in previously radiated patient. More than 50% patients are unresectable. Other options of salvage treatment like re-irradiation and chemotherapy are associated with poor response rates and high incidence of acute and late toxicity (Gr ≥3 toxicity 50–70%). SABR is a novel technology to deliver high dose of radiation to recurrent tumor with high precision. It yields high local control rates with less toxicity compared to conventionally fractionated radiation.

## Introduction

Locoregional failure accounts for approximately 40–60% of deaths and is the most common cause of death in locally advanced head and neck cancers (HNCs) despite improvements in multimodality care^1^^.^ Although salvage surgery is the most effective treatment modality, a large proportion of patients with recurrent HNC presents with unresectable recurrences and have poor outcome.^2^ Systemic chemotherapy and re-irradiation with conventional fractionation are frequently utilized in patients with recurrent HNC. However, these modalities yield poor survival and are also associated with high rates of acute and late toxicities.^3–5^ Issue of treatment related morbidity is of great significance in these patients with pre-existing late sequelae like dryness of mouth, varying degrees of dysphagia, aspiration and weight loss.

Currently, the criteria for selection of one treatment modality over the other in patients with recurrent, previously radiated and unresectable HNC are unclear. There are number of factors which can affect the selection of treatment like site of recurrence, volume of recurrent tumor, modality of previous treatment (Surgery or Radiation or both), time since previous treatment, operability and MIRI RPA class (Recursive Partitioning Analysis) etc. A significant proportion of patients present with poor performance status. A large multi-institutional study has reported distinct prognostic subgroups by means of RPA class.^6^ 2 years survival of MIRI RPA Class I, II and III patients was 61.9, 40.0 and 16.8% respectively in this study. No patient in MIRI RPA Class III experienced long-term survival and had a median survival of 8 months despite protracted re-irradiation with modern radiation techniques like IMRT.

Stereotactic ablative radiotherapy (SABR) differs from other modern radiation techniques like IMRT in that it delivers higher dose per fraction to a precise tumor volume with greater precision in localization and delivery of dose with stereotaxy. Since it exposes much smaller volumes of normal tissue to radiation, the normal tissue toxicities with SABR are usually less than those of 3D-conformal radiation and IMRT techniques. Another advantage of SABR is short treatment time of 1–2 weeks (for delivering typically five fractions) as compared to IMRT which is delivered over 6–7 weeks. Treatment duration is an important consideration in these patients with limited survival.

In this study we have evaluated the outcome of patients of recurrent and previously irradiated HNC patients treated with SABR and evaluated the factors which can affect patient outcome.

## Methods and materials

We analyzed 32 consecutive patients of recurrent head and neck cancer who were treated with re-irradiation with SABR over a period from 2014 to 2018. Study was approved by institutional review board (reference number RS/MSSH/SKT-2/ONCO/IEC/18–61). All the patients had a written informed consent. SABR was offered to patients who were deemed inoperable by a multidisciplinary tumor board. It was either done for locally recurrent disease or regional lymph node recurrence. The inclusion criteria included patients with recurrent or second primary HNC who had previously received radiation therapy as the definitive modality or in the post-operative setting. They were previously treated with conformal radiation with IMRT and IGRT. The dose of radiation delivered in radical radiation ± concurrent chemotherapy group was 66–70 Gy in 6 ½−7 weeks and 60–62 Gy in patients who received post-operative radiation. It included patients with clinically or radiologically well-discernible lesion. Patients had an ECOG PS of 0–2 and adequate hematological, hepatic and renal functions. Exclusion criteria included recurrent head and neck patients who had not received radiation previously and patients who had received prior radiation to a dose of <40 Gy EQD2.

Staging workup included history and physical examination, complete blood count, liver and kidney function test, chest X-ray, indirect laryngoscopy, direct laryngoscopy and biopsy. CT-positron emission tomography (CT-PET) and /or MRI head and neck region was done for better delineation of local extent of disease and also to rule out metastatic disease. Target delineation in each patient was confirmed by a radiologist.

### Treatment planning and delivery

**Figure 1.**
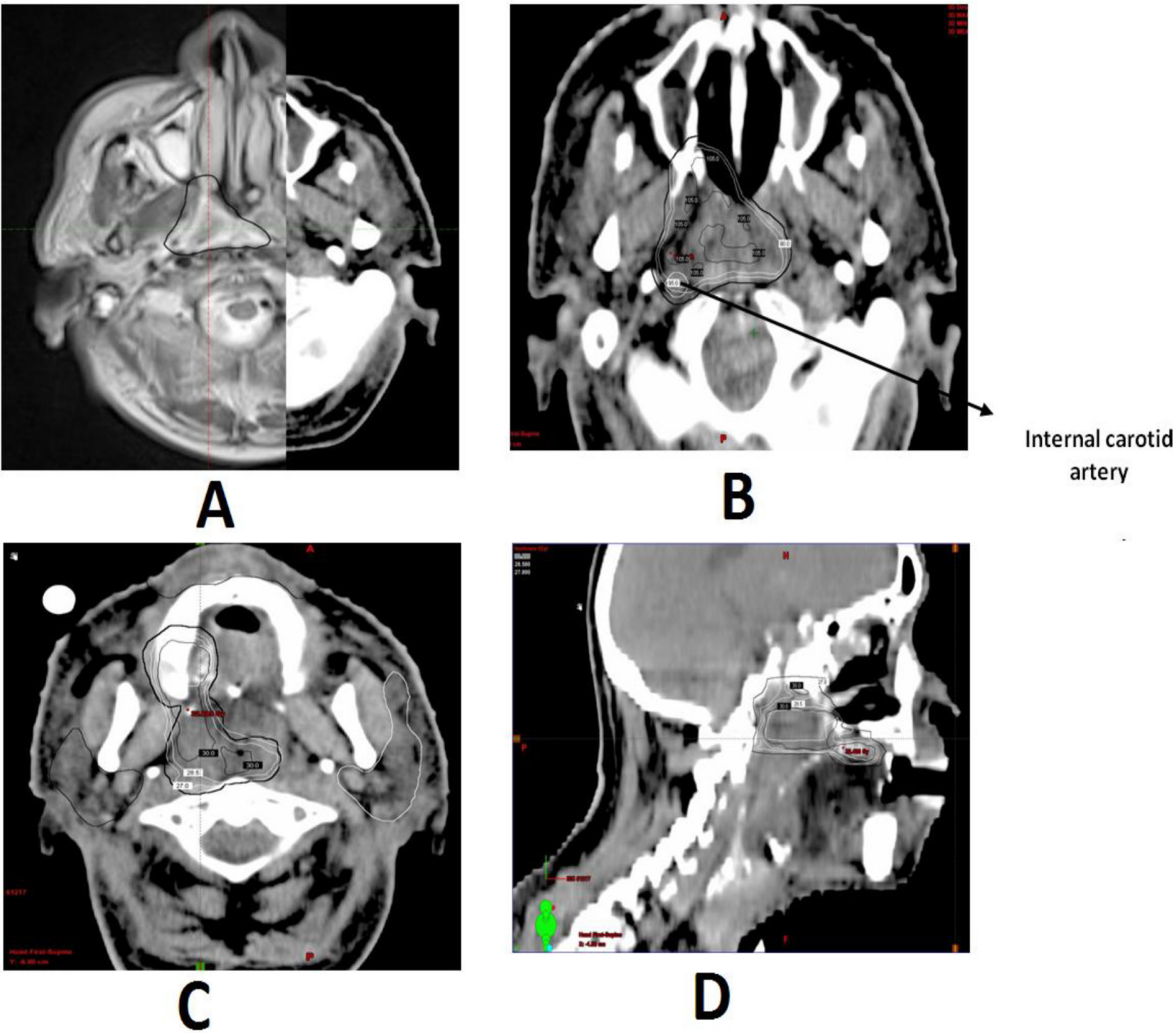
(A) Local recurrence in a patient of carcinoma nasopharynx (diagnosed in February 2011' and treated with chemoradiation) marked in black as delineated on fused CT-MRI scan. Figure 1B, (90%) Isodose with partial sparing of internal carotid artery covering tumor volume for a prescribed dose of 30 Gy/five fractions. Figure 1C,D, Two separate but adjacent GTV's covered in the prescribed dose as shown in axial and saggital views. Patient is alive and remains disease free 27 months after SABR. GTV, gross tumor volume; SABR, stereotactic ablative radiotherapy.

A customized three layered thermoplastic mask (Brainlab, Feldkirchen, Germany) was fabricated and simulation CT of the region of interest with intravenous contrast and ‘Exactrac’ CT localizer box was obtained with a 2 mm slice thickness. The target volume and organs at risk (OARs) were defined based on simulation CT images fused with magnetic resonance scan and or PET-CT scan. The gross tumor volume (GTV) was the clinically visible tumor and tumor defined in the imaging studies (Figure 1). A margin of 3 mm axially and 5 mm craniocaudially (cc) was added to the GTV for the planning target volume (PTV). Larger cc PTV margin was taken to account for greater setup uncertainties in (cc) direction.

OARs included spinal cord, mandible, larynx, carotids, skin, oral cavity and parotids. PRV margin was given to the spinal cord where the thecal sac was not separately contoured. The total dose and number of fractions were determined individually according to the tumor volume, prior dose of radiotherapy; local morbidity specially the health of skin and oral mucosa, time interval since previous radiation, and the ECOG performance status. Dose prescription ranged from 25 to 40 Gy/5 fractions. After the first four patients, no patient received a dose of less than 30 Gy in five fraction (Table 1). All the patients were planned by volumetric modulated arc therapy (VMAT) with Rapid arc (Varian Medical Systems, Palo Alto, CA). We used two full coplanar arcs for patients having tumor location near midline and two partial arcs and one non-coplanar arc for laterally positioned tumors with collimator position of 30-45^o^. All the plans were generated with high definition multileaf collimator (2.5 mm leaf thickness for centre 8 × 8 cm^2^ field followed by 5 mm leaf thickness) on Eclipse planning system (v. 10.0, Varian, Palo Alto, CA) (Figure 1). For lateralized tumors, skin was taken as OAR for skin sparing. Dose prescription was to the GTV ensuring that the PTV was encompassed by at least 80% isodose, with no more than 20% of PTV receiving >110% of the prescribed dose, no more than 2% of PTV receiving <93% of the prescribed dose, and no more than 5% of any normal tissue receiving dose in excess of 110% of the prescription dose. The PTV was cropped off the skin to allow for dose build-up. Plans were analyzed slice by slice for dose distribution and dose–volume histograms. Decay factors were used for OAR constraints which were determined individually taking previous radiation details into account and dose constraints suggested in AAPM TG 101. The carotid artery cumulative dose was limited to <120 Gy Equivalent dose (EQD2) and high priority was given to keep Dmax over carotid vessels below the prescription dose. Spinal cord cumulative dose was limited to 60 Gy (EQD2). All the patients were treated on Novalis Tx machine (Varian Medical Systems, Palo Alto, CA) with six-dimensional robotic couch. Image guidance on first day was carried out with both cone beam CT scan and ‘Exactrac system’ (Brainlab, Feldkirchen, Germany) and subsequently only with ‘Exactrac’ on each day of SABR delivery. Intrafraction snap verification was done for each arc and tolerance for correction was 1 mm. SABR was delivered for two consecutive days, 1 day break and then repeat for a total 5 fractions over 8 days.

**Table 1.** Demographic data and clinical characteristics (*n* = 32)

Variables	No. of Patients (%)
**Sex**	
Male	29 (90.6)
Female	03 (9.4)
**Age group (years**)	
30–40	02 (6.25)
41–50	05 (15.6)
51–60	13 (40.6)
61–70	08 (25)
>70	04 (12.5)
**Site of primary lesion**	
Oral cavity	19 (59.4)
Oropharynx	07 (21.9)
Hypopharynx /Larynx	02 (6.3)
Nasopharynx	03 (9.3)
Maxilla	01 (3.1)
**Primary treatment**	
Surgery and post operative radiation ( ± chemotherapy)	20 (62.5)
Radical radiation ( ± concurrent chemotherapy)	12 (37.5)
**MIRI RPA Class**	
Class II	20 (62.5
Class III	12 (37.5)
**Site of SABR**	
Neck node	12 (37.5)
Oral Cavity	10 (31.3)
Oropharynx	06 (18.8)
Nasopharynx	02 (6.3)
Oral and Neck	02 (6.3)
**GTV volume**	
<25 c.c	21 (65.6)
>25 c.c	11 (34.4)
**Dose of SABR**	
<30 Gy	04 (12.5)
30–35 Gy	22 (68.7)
36–40 Gy	06 (18.7)

GTV, gross tumor volume; RPA Class, recursive partitioning analysis class; SABR, stereotactic ablative radiotherapy.

### Response assessment and follow-up

Follow-up included physical examination and PET-CT/MRI scanning at 10–12 weeks post-SABR to assess the response. Subsequently, patients were followed with clinical examination at 3 months interval and or imaging in case of high index of suspicion of recurrence. Response was classified as complete response (CR), partial response (PR), progressive disease (PD) or stable disease (SD) as per RECIST/PERCIST criteria. Acute and late toxicity were recorded according to Common Terminology Criteria for Adverse Events (CTCAE) v. 3.0 (National Cancer Institute) and RTOG criteria respectively. Progression of lesion was defined as >20% increase in the sum of largest diameters of treated lesion (s) or appearance of new lesion. It was subsequently confirmed by biopsy. Local failure was defined as failure at the treated site and locoregional failure as failure within any head and neck site including lymphnodes. Distant failure was defined as failure outside head and neck region.

### Statistical analysis

Kaplan–Meier method was used for generating survival curves and to identify potential factors associated with local control (LC) and overall survival (OS). OS was calculated from the day of beginning of SABR till the time of death or last date of follow-up. Time to locoregional failure (LRF) was calculated as the time from starting SABR to last date of follow-up or date of disease recurrence within head and neck region. Univariate analyses (UVA) was performed with log rank test and factors found significant on UVA underwent multivariate analysis (MVA) with parsimonious modelling Cox regression model to identify independent risk factors. SPSS software v. 20.0 was used for statistical computation (SPSS Inc, Chicago, IL).

## Results

A total of 32 patients received SABR in the period January 2014–December 2018. Site of primary tumor is depicted in Table 1. Oral cavity was the predominant site (59.4%) of primary tumor. 20 patients had previously been treated with surgery and post-operative radiation (with or without concurrent chemotherapy) for their primary tumor and 12 patients had received radical radiation with or without concurrent chemotherapy. Median time to recurrence after primary treatment was 16 months (range 3–55 months). Patients were also classified according to MIRI RPA class (Table 1) as proposed by Ward et al.^6^ Class I included patients more than 2 years from their initial course of radiation with resected tumors; Class II included patients > 2 years with unresected tumors or those <2 years and without feeding tube or tracheostomy dependence and the remaining patients were classified as Class III. 14 patients (43.7%) had enteral feeding with either percutaneous endoscopic gastrostomy or Ryle’s tube, and 1 patient (2.9%) had tracheostomy at the time of recurrence.

Predominant site of recurrent tumor was neck node (12 out of 32 patients) followed by oral cavity, oropharynx and nasopharynx (Table 1). Contrast-enhanced MRI was available in 5 patients and CT-PET was available in 29 patients for GTV delineation. Mean GTV of the entire group was 28.14 c.c. (range 3.42 – 171 c.c.). The most frequently prescribed dose of SABR was 30–35 Gy in five fractions and was delivered to 68.7% patients (22 out of 32). six patients received a dose of 35–40 Gy and four patients received <30 Gy (Table 1).

**Figure 2.**
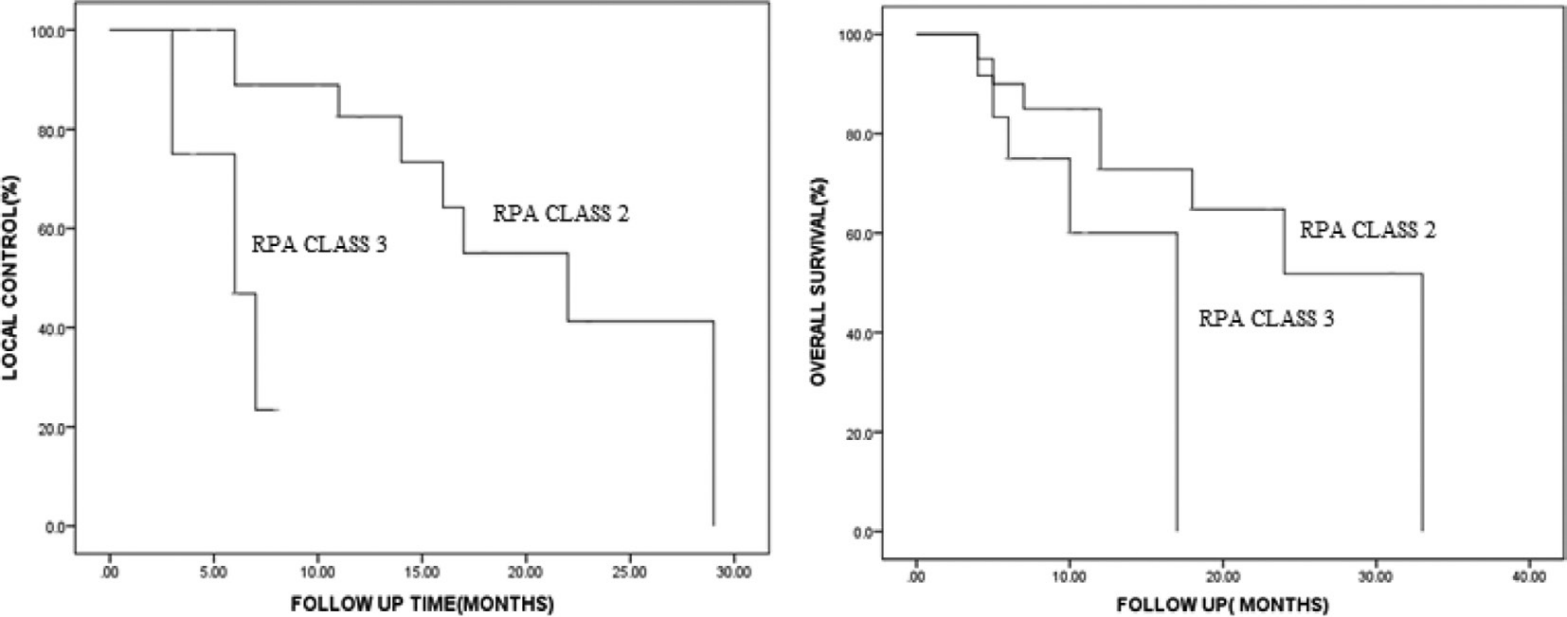
Kaplan–Meier graphs for A, LC—for MIRI RPA Class II and III and B, OS for RPA Class II, and III. LC, local control; OS, overall survival.

**Figure 3.**
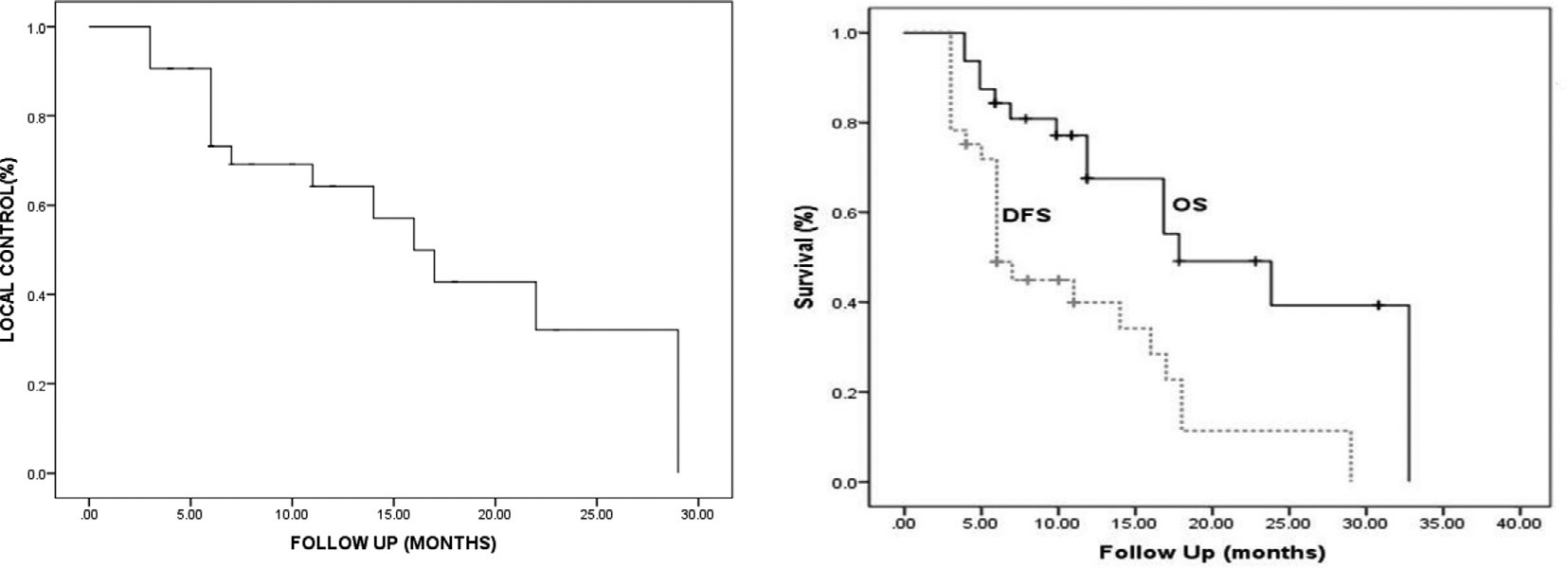
Kaplan–Meier graphs for A, LC; B, DFS and OS. DFS, disease free survival; LC, local control; OS, overall survival.

Median follow-up of the study after re-irradiation was 12 months (range 3–32 months). Local response was assessed 3 months after SABR. There was CR in 50% patients, PR 38.2%, SD 5.9% and PD in 5.9% patients. Median OS was 24 months of the entire cohort. LC and OS was 64.2 and 67.5% at 1 year and 32 and 39.5% at 2 years (Figure 2). 23 out of 32 patients (71.8%) developed recurrence/distant metastasis. Distant metastasis was the most frequent site of failure followed by local and local and regional recurrences (Table 2). Median LC and OS for RPA Class II was 22 and 33 months and for Class III was 6 and 17 months respectively (Figure 3).

**Table 2.** Patterns of recurrence (23/32)

	Number	%
Local recurrence	4	17.30%
Local and regional recurrence	4	17.30%
Distant metastases( ± Locoregional recurrence)	15	65.30%

Various prognostic factors were analyzed for LC and OS. For LC, on UVA, GTV volume, dose of SABR and time to re-irradiation (<1 year *vs* >1 year) were found to be statistically significant prognostic factors (Table 3). However, on MVA, dose of SABR and time to re-irradiation were significant prognostic factors for LC. For OS, GTV volume and dose of SABR came out statistically significant prognostic factors on both UVA and MVA (Table 3).

**Table 3.** Univariate and multivariate analysis of prognostic factors for local control and overall survival

Prognostic factor	**Local control** ‘*p*’ value	**Overall survival** ‘*p*’ value
**Univariate analysis**		
GTV volume (<=25 cc *vs* > 25 cc)	0.002	0.006
Biologically effective dose of SABR (<48 G_y10_ *vs* > 48 G_y10_)	0.011	0.004
Time to re-radiation (<1 year *vs* > 1 year)	0.001	0.150
Previous surgery *vs* no surgery	0.077	0.196
Local recurrence *vs* second primary	0.103	0.089
**Multivariate analysis**		
Biologically effective dose of SABR (<48 G_y10_ *vs* > 48 G_y10_)	0.002	0.034
Time to re-radiation (<1 year *vs* > 1 year)	0.01	NS
GTV volume (<=25 cc *vs* > 25 cc)	NS	0.041

GTV, gross tumor volume; SABR, stereotactic ablative radiotherapy.

### Treatment-related toxicity

Acute toxicity: At the time of SABR, 16 patients (50%) had pre-existing Grade 1 dysphagia, 3 patients (9.3%) Grade 2 dysphagia and 15 patients (46.8%) Grade 3 dysphagia. During treatment, dysphagia grade worsened from Grade 1 to Grade 2 in 6 patients and 1 patient had an improvement in dysphagia from Grade 2 to 1. 12 patients (37.5%) developed Grade 1 mucosal toxicity, 10 (31.3%) patients had Grade 2 and 9 patients (28%) had Grade 3 mucositis confined to high dose region. 21 patients had Grade 1 dermatitis and 10 patients Grade 2 dermatitis.

Late toxicity: 3 patients out of 32 (9.3%) had grade >3 late toxicity. Two patients on long-term nasogastric tube dependence prior to SABR, suffered episodes of aspiration pneumonia at 3 and 5 months post-SABR. However, both are alive with controlled treated lesion. One patient had Grade 3 mucosal reactions over the treated area and he succumbed to progressive disease, 4 months after SABR. One elderly gentleman (85 years old) had poor response to SABR and died 3 months later with progressive disease and aspiration.

## Discussion

Patients with recurrent and inoperable HNC present a challenging problem to both the treating oncologist and the patient. There are several options of treatment like re-irradiation with conventional fractionation ( ± chemotherapy), systemic chemotherapy, immunotherapy and palliative care.^3–5^ Decision making becomes complex due to high incidence of acute and chronic toxicity associated with these treatment options in patients previously treated with multimodality therapy of surgery, radiation and chemotherapy and short survival time.

Chemotherapy is frequently utilized in patients with recurrent HNC. However, it has response rates of 20–36%; median duration of response of 3–5.6 months and is associated with Grade 3 or 4 toxicity in 76–82% patients^3^.

Re-irradiation with conventional fractionation is another alternative for these patients. However, two landmark studies RTOG 9610 and 9911 achieved poor 2 years survival in the range of 15–26%.^4,5^ Further, re-radiation was associated with high rates of acute (63–78%) and late toxicities (22–37%) in patients with pre-existing late squealae like dryness of mouth and various degrees of dysphagia, aspiration and weight loss. It further adversely affected their quality of life.

Re-irradiation with modern radiation techniques like IMRT is better tolerated than older techniques like 3D-CRT.^6^ The risk of acute grade >3 complications of 22.1% and late grade >3 toxicity of 16.7% has been reported with IMRT.^7^ Patients with poor performance status and MIRI RPA Class III fair poorly with IMRT.^7^ In another study, 2 year cumulative incidence of grade >3 late toxicity was reported as 14.2%.^8^ This study also identified that MIRI RPA Class III patients were not ideal candidates for protracted chemoradiation regardless of resection status. In the present study, 37.5% patients were in MIRI RPA Class III and hence would have faired poorly with protracted radiation therapy.

SABR is highly conformal form of radiation therapy delivered with stereotactic precision to a well defined tumor volume with sharp fall-off dose to surrounding critical structures. It is technically challenging but has radiobiological advantage due to delivery of high dose per fraction. SABR may be a better therapeutic option in MIRI RPA Class III patients due to better sparing of surrounding normal tissues like uninvolved oral cavity, larynx, hypopharynx, mandible or even skin and mucosa adjacent to the recurrent lesion.^7^ LC and OS were found to be comparable with IMRT and SABR when a SABR dose of >35 Gy was delivered in MIRI RPA Class II patients.^9^ All the patients in this study were in MIRI RPA Class II and III. Median OS in the present study was 24 months which compares favorably with OS with re-irradiation with IMRT (16.5 months) as reported by Ward et al.^8^

Volume of recurrent tumor is an important parameter for deciding the suitability of SABR. In the present study, GTV of <25 c.c had better LC and OS with SABR on UVA, However, on MVA, only OS was statistically significant (Table 3). Volume of recurrent tumor was also found as significant prognostic factor in a study by Vargo et al.^9^ Bigger and more diffuse recurrence may be unsuitable for SABR.

There seems to be a dose–response relationship for SABR in recurrent HNCs. The present study showed statistically significant improved local control with a dose of >48 G_y10_ in five fractions as compared to <48 G_y10_ on UVA and MVA. OS benefit was also seen with higher dose of SABR on both UVA and MVA. Similar observation has been reported in a systemic review in the report of the AAPM working group.^9^ A dose of 30–45 Gy (in five fractions) was associated with superior LC and OS as compared with dose <30 Gy. In the present study, there was improvement in both LC and OS on MVA with higher SABR dose. All the patients in the present study received SABR with VMAT, since VMAT plans were found to be superior to IMRT plans. Two arc plans were better than single arc plans as also reported by Eugenio et al.^10^

Acute toxicity reported with re-irradiation with IMRT ranges from 16 to 22%.^7^ In a comparative analysis, new grade >3 acute toxicity occurred in 16.6% of IMRT patients and 11.7% of SABR patients which was not statistically significant.^7^ However, grade >4 acute toxicity (fistula development, intensive care admission and life threatening bleeding) was more common with IMRT than SABR (5.1% *vs* 0.5%; *p* < 0.01).^7^ Radiation dose to carotid vessels should be minimized to prevent carotid -blowout syndrome with SABR. Some of the factors reported to potentially increase the risk of carotid blowout include >180˚ carotid involvement, carotid dose >100% of prescription dose, and D_0.1cc_ of carotid vessels > 39.4 Gy^11^. In the present study, treatment-related Grade 3 acute mucosal toxicity occurred in 28.1% patients mainly confined to high dose region of SABR. No other treatment related acute grade >3 toxicity was seen. Late toxicity occurred in 9.3% of patients in this study and it compares favorably with late toxicity associated with IMRT which has been reported to range from 16 to 66% and it increased with time.^12,13^ Low rates of toxicity in the present study could also be due to no patient of Larynx/hypopharyngeal site received SABR. Also, skin toxicity was minimized by editing PTV for skin dose build-up. One of the limitation of the study can be that we did not analyze toxicity in relation to target volume.

The role of systemic therapy is being explored with SABR since distant metastasis remains a significant problem in this patient population. In the present study, 65.3% patients developed distant metastasis and was the predominant site of failure (Table 2). It warrants exploration of novel systemic agents to reduce the risk of distal metastasis. Few studies have reported the safety and efficacy of addition of concurrent Cetuximab with this approach.^14^ Another exciting aspect of SABR is its role as “immunogenitor”. Hypofractionated radiation therapy like SABR is known to augment immune response primarily by releasing tumor antigens and upregulation of major histocompatibility complex on the surface of tumor cells leading to activation of T-cell mediated response. The radiation-induced immune-mediated response not only controls the locally radiated site but also the nearby field (“Bystander Effect”) and distant metastatic sites (“Abscopal Effect”).^15,16^

Preclinical work in animal models of melanoma, breast cancer, and colorectal cancer combining radiation with check point blockade has shown favorable responses.^17^ It is prudent to combine SABR or hypofractionated radiation with immune checkpoint inhibitors to further enhance the immune-mediated responses. Currently, Keystroke trial is underway looking at whether addition of pembrolizumab to SBRT will improve progression free survival for patients with recurrent or new second primary head and neck squamous cell carcinoma.^18^ However, these newer approaches with SABR are work in progress and need longer follow-up to further define their role in the current management of recurrent HNC.

## Conclusion

Thus, in recurrent HNC, SABR yields LC and survival which compares favorably with wide field irradiation with conformal techniques like IMRT. SABR is much better tolerated with fewer treatment related morbidities and short treatment time. It may be a preferred treatment option in patients with limited volume recurrence. In view of high incidence of distant metastasis, novel systemic therapies need to be explored for improvement in overall survival in combination with SABR.
